# Differential effects of elevated cholesterol and fatty acids on endothelial function: Mechanisms contributing to atherosclerosis development

**DOI:** 10.6026/973206300213026

**Published:** 2025-09-30

**Authors:** Nanda Kumar C., Sampath Kumar P., Aysha Asath, Kalpeshkumar Amrutlal Parmar, Shiv darshan Rao, Anil Kumar Medanki

**Affiliations:** 1 Department of General Medicine, VEL'S Medical College, Chennai, India; 2 Department of Emergency Medicine, Chettinad Superspeciality Hospital/Chettinad Hospital and Research Institute, Kelambakkam, Chennai, India; 3 Department of General Surgery, Dharmsinh Desai University, Nadiad, Gujarat, India; 4 Department of OMFS, Teerthanker Mahaveer Dental College & Research Centre, Moradabad, Utter Pradesh, India; 5 Department of Anatomy, SRMC & GH, Nandyal, Kurnool, Andhra Pradesh, India

**Keywords:** Endothelial dysfunction, LDL cholesterol, fatty acids, nitric oxide, oxidative stress, atherosclerosis

## Abstract

The impairment of endothelial function occurs through LDL cholesterol and saturated/ trans fatty acids which cut down NO production while rising ROS levels and starting NF-κB signaling processes. The activity of glycolysis becomes blocked because palmitic acid triggers PKM2-C31 palmitoylation which leads to increased damage. Among the tested fats trans fats elaidic and linoelaidic created the most damaging oxidative reactions. The monounsaturated fat oleic acid promoted both elevated NO production and decreased inflammation in cells. Thus, we show that different lipids activate unique biological mechanisms toward the production of atherosclerosis.

## Background:

Effective endothelial function represents the foundation of vascular health and the starting point for both atherosclerosis development and the progression of cardiovascular disease [1]. The endothelium is now recognized as a dynamic organ that plays a central role in maintaining vascular homeostasis by regulating vascular tone, inflammation, coagulation, and vascular growth [2]. A healthy endothelium relies on nitric oxide (NO) production to induce vasodilation, inhibit platelet aggregation, prevent leukocyte adhesion, and suppress smooth muscle cell proliferation [3]. There is a well-established association between dyslipidemia-particularly elevated low-density lipoprotein cholesterol (LDL-C) and abnormal fatty acid distribution and endothelial dysfunction, which accelerate the progression of atherosclerosis [4, 5]. Evidence suggests that cholesterol levels have a continuous relationship with endothelial function, where even modest elevations within the normal range can reduce vasodilatory capacity [6]. Conversely, small reductions in cholesterol levels significantly improve endothelial function, highlighting the sensitivity of endothelial cells and the cardiovascular benefits of lipid-lowering interventions [7]. LDL-C contributes to endothelial injury through multiple mechanisms. Once oxidized, LDL promotes the transformation of monocytes into foam cells, initiating atherosclerotic plaque formation [8]. Oxidized LDL interferes with endothelial nitric oxide synthase (eNOS) translocation to caveolae in the plasma membrane, thereby reducing NO production [9]. In addition, oxidized LDL increases reactive oxygen species (ROS) formation, which further decreases NO bioavailability through peroxynitrite production [10]. Oxidative stress activates nuclear factor-kappa B (NF-κB); initiating pro-inflammatory signaling that perpetuates endothelial dysfunction [11]. Beyond cholesterol, fatty acid composition also influences endothelial health. Different fatty acids exert overlapping, and sometimes opposing, effects on endothelial function [12]. Experimental studies show that saturated fatty acids, particularly palmitic acid, activate inflammatory pathways, increase ROS production, and impair endothelial function [13]. Palmitic acid has also been shown to induce palmitoylation of pyruvate kinase M2 (PKM2), disrupting endothelial glycolysis and contributing to cardiovascular complications [14]. Trans fatty acids, such as elaidic and linoelaidic acids, are similarly harmful, promoting NF-κB activation, reducing NO availability, and increasing superoxide production in endothelial cells [15]. In contrast, monounsaturated fatty acids (MUFAs) demonstrate protective vascular effects. Oleic acid, abundant in olive oil, has been shown to suppress cytokine-induced expression of vascular cell adhesion molecule-1 (VCAM-1) and other adhesion molecules in endothelial cells, thereby reducing monocyte attachment during the early stages of atherosclerosis [16, 17]. This protective action may be partly explained by the replacement of saturated fatty acids in cell membrane phospholipids with oleic acid, which enhances endothelial resilience [17]. Therefore, it is of interest to investigate the relationship between lipid profile components, specific fatty acids, and endothelial function in order to better understand their roles in the development and prevention of atherosclerosis.

## Materials and Methods:

The laboratory obtained Human Umbilical Vein Endothelial Cells (HUVECs) from Lonza (Basel Switzerland) which they cultured inside Endothelial Growth Medium (EGM-2 Lonza) combined with 10% fetal bovine serum (FBS) containing endothelial cell growth supplements at 37°C in a humidified atmosphere with 5% CO2. Experimentation used HUVEC cell samples from the third to sixth passage. Human LDL obtained from Sigma-Aldrich in St. Louis was incorporated into serum-free medium to expose HUVECs to concentrations of 50, 100, 150, and 200 µg/ml for 24-hour incubation. LDL oxidation happened through 5 µM CuSO4 incubation at 37°C for 24 hours followed by PBS dialysis. Detection of oxidation occurred through thiobarbituric acid reactive substances measurement. The cell cultures received fatty acid treatments with sodium salts of palmitic acid (C16:0) and elaidic acid (trans-C18:1) and linoelaidic acid (trans-C18:2) and transvaccenic acid (trans-C18:1) and oleic acid (cis-C18:1) at a concentration of 100 µM for 24-72 hours (all materials from Sigma-Aldrich). Staff researchers conducted an albumin-attachment reaction between fatty acids and fatty acid-free bovine serum albumin (BSA) at 4:1 molar levels before these solutions entered culture medium. Scientists measured nitric oxide production via the fluorescent probe 4,5-diaminofluorescein diacetate (DAF-2DA obtained from Cayman Chemical, Ann Arbor, MI). After washing the cells with PBS they received 5 µM DAF-2DA solutions for fluorescence detection over a period of 30 minutes at 37°C. The microplate reader evaluated fluorescence with excitation at 485 nm and emission at 535 nm following insulin treatment with 100 nM or application of 1 µM calcium ionophore A23187. Cell lysate separation proceeded by SDS-PAGE before proteins were subjected to immunoblotting with phospho-eNOS (Ser1177) and total eNOS and β-actin antibodies purchased from Cell Signaling Technology in Danvers, MA. Laboratory measurements of ROS produced inside cells utilized the cell membrane penetrating agent 2', 7'-dichlorodihydrofluorescein diacetate (H2DCFDA, Invitrogen, Carlsbad, CA). Cells incubated with 10 µM H2DCFDA for 30 minutes had washes before the fluorescence was measured with 485 nm excitation and 535 nm emission wavelengths. The lucigenin-enhanced chemiluminescence method served to determine the levels of superoxide in samples. The buffer solution for cell lysis consisted of 20 mM HEPES at pH 7.4 together with 10 mM KCl and protease inhibitor additives. The assay involved lucigenin at 5 µM concentration together with NADPH at 100 µM which were added to the cell lysate solution for measurement of chemiluminescence using a luminometric instrument. A surface enzyme immunoassay technique determined the levels of adhesion molecules (VCAM-1, ICAM-1, E-selectin). The experimental procedure started by fixing cells with a solution containing 0.1% glutaraldehyde before blocking them with 1% BSA then adding primary antibodies followed by incubation with HRP-conjugated secondary antibodies. The tetramethylbenzidine solution functioned as an indicator for the colorimetric reaction which allowed for absorbance detection at 450 nm. The research analyzed NF-κB activation through electrophoretic mobility shift assay (EMSA) and IκBα phosphorylation and degradation determination through immunoblotting methods. The acyl-biotin exchange method was used for evaluating PKM2 palmitoylation. A whole cell extract obtained after losing the cells went through N-ethylmaleimide treatment to block free thiols. The experimental procedure included protecting palmitoylated proteins with HPDP-biotin after subjecting them to hydroxylamine treatment. The detection of PKM2 occurred through immunoblotting after protein binding to streptavidin-agarose beads utilizing biotin-labeled proteins. Each experiment consisted of three duplicate tests as part of at least three separate experimental rounds and the data evaluation included mean values and standard deviation (SD) measurements. The research team conducted their statistical tests with GraphPad Prism 9.0 installed on computers from GraphPad Software in San Diego, California. Student's t-test analyzed differences between two groups and one-way ANOVA followed by Tukey's post-hoc test performed the comparisons among multiple groups. Regulation of dose-response effects occurred through regression analysis evaluation. The analysis revealed statistical significance when the p-value reached below 0.05.

## Results:

Exposure of HUVECs to increasing concentrations of LDL-C for 24 hours resulted in a dose-dependent impairment of endothelial function. As shown in Table 1 (see PDF), nitric oxide production in response to insulin stimulation significantly decreased with increasing LDL-C concentrations. At 150 µg/ml, LDL-C reduced NO production by 42.3% compared to control (p<0.001). This reduction was accompanied by a significant decrease in eNOS phosphorylation at Ser1177, which is essential for eNOS activation. At the highest concentration tested (200 µg/ml), eNOS phosphorylation was reduced by 59.1% (p<0.001). Data are presented as mean ± SD from five independent experiments. *p<0.05, **p<0.01, ***p<0.001 vs. control. Concurrent with decreased NO production, LDL-C dose-dependently increased ROS generation, with a 3.1-fold increase at 200 µg/ml (p<0.001). The expression of VCAM-1, a key adhesion molecule involved in monocyte recruitment, also increased with rising LDL-C concentrations, showing a significant 2.9-fold increase at 200 µg/ml (p<0.001). Phosphorylation of IκBα, indicating NF-κB activation, showed a similar dose-dependent increase, suggesting activation of inflammatory pathways by LDL-C. Regression analysis revealed a strong negative correlation between LDL-C concentration and NO production (r = -0.91, p<0.001), and a strong positive correlation between LDL-C concentration and ROS production (r = 0.89, p<0.001), confirming the dose-dependent relationship between LDL-C exposure and endothelial dysfunction. The effects of different fatty acids on endothelial function markers after 24 hours of exposure are presented in Table 2 (see PDF). Saturated and Trans fatty acids exhibited detrimental effects on endothelial function, while oleic acid demonstrated protective effects.

Data are presented as mean ± SD from five independent experiments *p<0.05, **p<0.01, ***p<0.001 vs. control. The most prevalent saturated fatty acid named Palmitic acid decreased both NO production by 44.7% (p<0.001) and eNOS phosphorylation by 56.7% (p<0.001). An increase of 2.8-fold ROS (p<0.001) and 2.4-fold superoxide (p<0.001) accompanied these impacts. The research indicates that palmitic acid treatment resulted in a 2.9-fold increase of PKM2 palmitoylation (p<0.001) which may help explain how palmitic acid causes endothelial dysfunction. The Trans fatty acids elaidic acid and linoelaidic acid produced heightened impacts by causing a 54.8% decrease in NO production while raising ROS production by 3.5-fold (p<0.001). TNF-α-induced VCAM-1 expression together with increased IKKβ activity strongly indicated inflammatory pathway activation through both Tran's fatty acids. The endothelial effects of transvaccenic acid derived from ruminant sources remained insignificant compared to the control conditions since this Trans fatty acid showed no variations in NO production or ROS generation or inflammatory marker expression. The endothelial protective action on function was demonstrated by monounsaturated fatty acid oleic acid most prominently. The use of oleic acid resulted in a 38.7% (p<0.01) increase in NO production together with a 27.3% (p<0.05) increase in eNOS phosphorylation and a 30% (p<0.05) reduction in ROS production. The use of oleic acid as a treatment agent demonstrated substantial anti-inflammatory properties because it reduced VCAM-1 expression by 43.2% (p<0.001) and decreased IKKβ activity by 40% (p<0.01). Cells exposed to oleic acid for seventy-two hours showed substantial fatty acid changes resulting in increased oleic acid at twenty-three percent while saturated fatty acids decreased at eighteen percent (stearic acid and palmitic acid) and polyunsaturated acids remained consistent. Saturated and trans fatty acids started influencing NO production and ROS generation between 6 hours after initial exposure and continued to enhance their effects at the 24-hour mark. The protective actions of oleic acid need 24 hours of contact to reach maximum efficacy because oxidative lipid membrane modifications probably play a role in its protective outcome.

## Discussion:

The research establishes detailed proof of how LDL cholesterol along with particular fatty acids impacts endothelial function by demonstrating separate yet combined mechanisms involved in atherosclerosis development. Endothelial function decreases proportionally to LDL cholesterol exposure but different fatty acids influence endothelial cells based on their molecular properties and fat saturation level [6]. Our research supports previous clinical findings by showing that elevated cholesterol even within normal ranges yet reduces endothelial vasodilation through a dependent negative impact on NO production and eNOS phosphorylation. The data indicate a gradual link between cholesterol amounts and vascular health status which confirms the principle that lower LDL cholesterol levels lead to improved vascular health [7]. Our research shows that increasing LDL cholesterol amounts leads to higher ROS development levels and NF-κB signaling-based inflammatory mechanisms which support earlier study results [10, 11]. The research demonstrates that oxidative stress functions as the main mechanism which drives endothelial dysfunction when exposed to LDL. Higher ROS production and lower NO bioavailability exist in direct relation and point to ROS-mediated NO scavenging as the main process through which LDL damages endothelial function. Research confirms that reactive oxygen species reduce NO bioavailability during endothelial dysfunction within hypercholesterolemia [5, 10]. The experimental findings back previous research which shows cholesterol affects how eNOS localizes within caveolae structures and thereby modifies its operational capacity [9]. The study demonstrates that saturated/trans fatty acids generate opposing effects to monounsaturated fatty acids regarding their impact on endothelial function. The majority of saturated fatty acids found in plasma plasma named palmitic acid demonstrated powerful adverse effects on endothelial function through its inhibitory actions on NO synthesis and increased oxidative stress. Research data matches earlier studies which demonstrated free fatty acids cause hindrance to eNOS activation after insulin exposure via IKKβ-dependent routes [12].

The research findings uncover significant PKM2 palmitoylation rates after palmitic acid exposure which agrees with current evidence supporting PKM2 post-translational modifications as a cause of endothelial injury and cardiovascular dysfunction [14]. The endothelial dysfunction triggered by trans fatty acids elaidic and linoelaidic acids was worse than saturated fatty acids because these acids lowered NO production more severely and raised inflammatory markers at higher levels. The study results confirm medical observations linking trans-fat intake to higher cardiovascular risks and multiple research reports finding trans isomers to activate NF-κB activity and reduce NO production in vascular cells [15]. The study results showed no considerable impact on endothelial function from transvaccenic acid a trans fatty acid that mainly comes from ruminant sources. This matches earlier research that demonstrates different trans fatty acid variations found in industrial production versus ruminant sources [18, 20]. The primary significant result detected through our research shows that oleic acid functions as a protective agent against endothelial dysfunction. The intake of oleic acid strengthened NO synthesis and reduced oxidative responses as it diminished inflammatory responses by decreasing VCAM-1 protein expression and reducing IKKβ enzyme activity. Scientific data shows that oleic acid reduces cytokine-mediating expression of adhesion molecules in endothelial cells as previous research demonstrated [16]. The protective course of oleic acid correlates with its time-dependent behavior while simultaneously modifying fatty acid composition within phospholipids which results in membrane fluidity alterations or lipid raft compositional changes serving as protective mechanisms. The examination results establish significant knowledge about the intricate association between lipid irregularities and atherosclerotic development [17].

The molecular mechanisms through which elevated LDL cholesterol and detrimental fatty acids lead to oxidative stress and inflammation appear different even though both conditions use similar biochemical pathways. High LDL cholesterol levels damage endothelial function mainly through the creation of oxidized molecules and disturbances of eNOS positioning and activity and specific fatty acids transform membrane structures and modify protein attachments and initiate signaling pathways up to NF-κB activation [19]. Several factors limit the validity of this research finding. The laboratory-based experimental design fails to reproduce the complete *in vivo* setting where various elements operate to affect endothelial operations. Our investigation focused on individual fatty acids yet dietary patterns normally combine these acids which might lead to combined favorable or unfavorable reactions. This analysis used HUVECs as research subjects even though arterial endothelial cells show potential divergence in their responses during atherosclerosis. Research should overcome these constraints by conducting studies that analyze cholesterol along with fatty acids on arterial endothelial cells and animal models with atherosclerosis.

## Conclusion:

We show that LDL cholesterol and saturated/trans fatty acids cause endothelial dysfunction through oxidative damage and decreasing nitric oxide yet demonstrates oleic acid protects the cells. The specific mechanisms involving lipids play an essential role in creating conditions for the development of atherosclerosis. Thus, we show nutritional approaches which focus on lowering cholesterol content while establishing beneficial fatty acid patterns for better vascular health outcomes.
